# Palliative Care in Heart Failure: Challenging Prognostication

**DOI:** 10.7759/cureus.18301

**Published:** 2021-09-26

**Authors:** Inês Egídio de Sousa, Ana Pedroso, Beatriz Chambino, Marta Roldão, Fausto Pinto, Renato Guerreiro, Inês Araújo, Célia Henriques, Candida Fonseca

**Affiliations:** 1 Internal Medicine Department, Hospital São Francisco Xavier, Lisbon, PRT; 2 Heart Failure Clinic, Department of Internal Medicine, Hospital São Francisco Xavier, Lisbon, PRT

**Keywords:** heart failure, palliative care, mortality, prognostication, maggic score

## Abstract

Heart failure (HF) is a chronic progressive disease with high morbimortality and poor quality of life (QoL). Palliative care significantly improves clinical outcomes but few patients receive it, in part due to challenging decisions about prognosis.

This retrospective study, included all patients consecutively discharged from an Acute Heart Failure Unit over a period of one year, aiming to assess the accuracy of the Meta‐Analysis Global Group in Chronic Heart Failure (MAGGIC) score in predicting mortality. Additionally, predictors of death at one and three years were explored using a multivariate regression model.

The MAGGIC score was useful in predicting mortality, without significant difference between mortality observed at three-years follow-up compared with a mortality given by the score (p=0.115). Selected variables were statistically compared showing that poor functional status, high New York Heart Association (NYHA) at discharge, psychopharmacs use, and high creatininemia were associated with higher mortality (p<0.05).

The multivariate regression model identified three predictors of one-year mortality: psychopharmacs baseline use (OR=4.110; p=0.014), angiotensin-converting enzyme inhibitors/angiotensin receptor blocker (ACEI/ARB) medication at discharge (OR=0.297; p=0.033), and higher admission's creatinine (OR=2.473; p=0.028). For three-year mortality outcome, two variables were strong independent predictors: psychopharmacs (OR=3.330; p=0.022) and medication with ACEI/ARB at discharge (OR=0.285; p=0.018). Models’ adjustment was assessed through the receiver operating characteristic (ROC) curve. The best model was the one-year mortality (area under the curve, AUC 81%), corresponding to a good discrimination power.

Despite prognostication, when setting goals of care an individualised patient-centred approach is imperative, based on the patient’s objectives and needs. Risk factors related to poorer outcomes should be considered, in particular, higher NYHA at discharge which also represents symptom burden. Hospitalisation is an opportunity to optimize global care for heart failure patients including palliative care.

## Introduction

Heart failure (HF) is a chronic and progressive disease and its prevalence in developed countries is around 12% in population aged 65 years and over with a trend to increase [1,2]. It is an important healthcare problem, associated with high morbidity and mortality rates as well as poor quality of life (QoL) [3-6]. Patients living with HF often struggle with poorly controlled symptoms, such as dyspnoea and fatigue, and high rates of depression, anxiety, and psychosocial stress [7].

Evidence suggests that a palliative approach in HF significantly improves patient outcomes, including symptom control and mental health, leading to decreased hospital admissions, decreased mortality, and reduced healthcare costs [7]. Palliative care is the active holistic care of individuals across all ages with serious health-related suffering due to severe illness and especially of those near the end of life. It aims to improve the QoL of patients, their families, and their caregivers [8]. According to World Health Organization (WHO), European Society of Cardiology (ESC), and Portuguese Palliative Care Program, palliative care should be available and early integrated into the routine care for patients with HF alongside disease-modifying treatment, and not only in the last days of life [9-11].

Although it is generally recognized that HF is a serious condition and equivalent to malignant disease in terms of symptom burden and mortality, only a comparatively small number of HF patients receive specialist palliative care [12-15]. A referral is not only lower than in other specialties but also late in the course of disease [16].

Although all patients with HF benefit from palliative care, not all patients require a specialised approach, particularly important in the advanced HF population who have a poor prognosis and high symptom burden [17]. Physicians find it difficult to identify which HF patients should be integrated in specialised palliative care as there is no uniform criteria for referral and prognostication is challenging [15,16]. During hospitalisation, patients should be routinely screened for palliative care needs and patients in end of life should be identified [18].

Objective risk scores incorporating clinical demographics, laboratory variables, imaging data, and hospitalisation history can help identify advanced HF patients who benefit from specialised palliative care [17]. Several prognostic risk scores have been developed and numerous variables have been associated with mortality and re-hospitalisation in heart failure [19]. The Meta‐Analysis Global Group in Chronic Heart Failure (MAGGIC) investigators developed an HF stratification risk model, clinically attractive as it is simple to use, including routinely collected variables, that was shown to be highly effective at predicting one-year and three-year mortality in patients with HF [20].

This study aims to assess the accuracy of the MAGGIC score in predicting the mortality of hospitalised patients with acute HF, as well as identifying the impact of other variables on the mortality of these patients, in order to recognize high-risk HF patients for early referral to specialised palliative care.

## Materials and methods

This is a single-centre cohort study including all adults consecutively discharged from an Acute HF Unit of a tertiary, University hospital in Portugal, over a period of one year (2016). 

The study consisted of a retrospective analysis of individual electronic medical records. Patients were eligible for inclusion if they were hospitalised for decompensated HF according to ESC guidelines criteria and excluded when there were insufficient data. Written informed consent was obtained from all subjects or their legally authorized representatives. This study was exempt from IRB review in accordance with the exemption criteria laid down by our Hospital Ethics Committee.

The entire study was purely observational in design; no interventions were applied as part of the study protocol.

The primary objective was to assess the accuracy of the MAGGIC score in predicting the three-year mortality of hospitalised patients with acute HF. The MAGGIC score (composed of 13 clinical variables) was applied to all patients, differentiating them into six groups according to risk score: group 1 (score 0-16), group 2 (score 17-20), group 3 (score 21-24), group 4 (score 25-28), group 5 (29-32), and group 6 (score 36+). Observed mortality for each group at three years follow-up was compared to expected three-year mortality when using the MAGGIC score.

Secondary objectives were: (1) characterise patients with a diagnosis of HF in accordance with ESC guidelines, consecutively discharged from an Acute HF Unit of a central tertiary hospital, in a period of one year - selected variables were analysed and compared in terms of impact on mortality at one-year and three-years follow-up; (2) identify one-year and three-years predictors of mortality in HF patients, in order to identify variables that can help identify high-risk HF patients for early referral to specialised palliative care - a multivariate logistic regression was performed for two outcomes (one-year mortality and three-years mortality).

Statistical description

Subject demographics and baseline characteristics were summarised. All continuous variables were described using descriptive statistics, including the number of observations (n), mean, standard deviation (SD), median, minimum and maximum. All categorical variables were summarised using the number and percent of subjects. 

Patient baseline characteristics and treatments were statistically compared: (1) for continuous variables, normality was tested with Shapiro-Wilk test, using Q-Q plots and histograms; (2) differences between continuous variables were assessed using Independent two-sample* *t-test or Mann-Whitney-U test, depending on normality, whereas the Chi-squared test (χ^2^) was used for categorical values; (3) Wilcoxon Signed Rank Test was used for paired data (for comparing observed real mortality at three-years follow-up with the expected three-year mortality given by the MAGGIC score).

A logistic regression multivariate analysis was performed for each outcome (one-year mortality and three-year mortality). The multivariate model was constructed using forward stepwise regression based on the Likelihood Ratio. The following criteria for including a variable in the multivariate regression model were used: (1) bibliographic search and clinical relevance for selection of potential candidates with known clinical importance; (2) a univariate regression analysis was conducted on all variables and a p-value lower than 0.25 was defined for inclusion in the multivariate model. Basic assumptions for logistic regression were assured. A p-value of 0.05 was used to retain each variable in the multivariate model. The Receiver Operating Characteristic (ROC) analysis was used to generate C-statistics and assess model performance.

The following data were extracted for all patients: age, gender, functional status previous to hospitalisation, New York Heart Association (NYHA) functional class, specific comorbidities (current smoker, diabetes mellitus (DM), chronic obstructive pulmonary disease (COPD), and dementia), specific medication use (beta-blockers, ACEI/ARB and psychopharmacs), systolic blood pressure (SBP), ejection fraction (EF), body mass index (BMI), creatininemia at admission, time of HF first diagnosis.

Statistical analyses were performed using SPSS version 25 (IBM Corp., IBM SPSS Statistics for Windows, Version 25.0. Armonk, New York: IBM Corp).

The transparent reporting of a multivariable prediction model for individual prognosis or diagnosis (TRIPOD) and strengthening the reporting of observational studies in epidemiology (STROBE) statements were generally followed for the elaboration of this report.

## Results

Subject demographics and baseline characteristics are summarised in Tables 1-2.

**Table 1:** Continuous variables of subjects demographics. BMI: body mass index; SBP: systolic blood pressure.

	N		Mean	Median	SD	Minimum	Maximum
	Valid	Missing					
Age	116	0	74.01	76.5	10.975	42	98
Height (m)	83	33	1.655	1.65	0.1132	1.4	2
Weight (kg)	83	33	73.963	71	18.3491	40	133
BMI (kg/m^2^)	116	0	27.527	26.71	5.4642	14	46
SBP (mmHg)	116	0	147.11	142.5	33.97	84	240
Creatinine level (mg/dL)	116	0	1.338	1.22	0.60274	0.54	4.59
Ejection fraction (%)	116	0	43.7	45	10.851	15	62

**Table 2:** Categorical variables of subjects demographics. NYHA: New York Heart Association; DM: diabetes mellitus; COPD: chronic obstructive pulmonary disease; ACEI/ARB: angiotensin-converting enzyme inhibitors/angiotensin receptors blockers.

Gender	47.4% male (N=55)	52.6% female (N=61)			
Previous functional status	79.3% Independent (N=92)	19.0% Partial dependency (N=22)	1.7% Total dependency (N=2)		
NYHA class at admission	0.9% Class II (N= 1)	27.6% Class III (N= 32)	71.6% Class IV (N= 83)		
NYHA class at discharge	16.5% Class I (N= 19)	68.7% Class II (N= 79)	13.9% Class III (N= 16)	0.9% Class IV (N= 1)	0.9% Missing (N=1)
Current smoker	88.8% non-smoker (N= 103)	11.2% Smoker (N=13)			
DM	54.3% No DM (N=63)	45.7% with DM (N=53)			
COPD	85.3% No COPD (N=99)	14.7% with COPD (N=17)			
Dementia	91.4% No dementia (N=106)	8.6% with dementia (N=10)			
Beta-blockers at discharge	30.2% No beta-blockers (N=35)	69.8% with beta-blockers (N=81)			
ACEI/ARB medication at discharge	34.5% No ACEI/ARB (N=40)	65.5% with ACEI/ARB (N=76)			
Use of psychopharmacs	63.8% No psychopharmacs (N=74)	36.2% with psychopharmacs (N=42)	

The MAGGIC score was applied to all patients (composed of 13 clinical variables), differentiating them into six groups according to risk score: group 1 (score 0-16), group 2 (score 17-20), group 3 (score 21-24), group 4 (score 25-28), group 5 (29-32), and group 6 (score 36+). Observed mortality for each group at three-years follow-up was compared to expected three-year mortality when the MAGGIC score is applied. A Wilcoxon signed-rank test determined that there was not a statistically significant median difference between real mortality compared to the expected three-year mortality given by MAGGIC score (p = 0.115; Table 3). 

**Table 3:** Wilcoxon signed-rank test comparing the real three-year mortality and the expected three-year mortality calculated by MAGGIC score.

Median real three-year mortality	Median expected three-year mortality	p-value
33.5	29.5	0.115

 Patient baseline characteristics and treatments were statistically compared (Tables 4-5). For the one-year mortality outcome, there was a significant difference in creatininemia at admission between groups [U=1243, p=0.014]. The variables associated with one-year mortality were previous functional status: [χ^2^(2) = 9.978; p=0.007], NYHA class at discharge [χ^2^(3) = 12.143; p=0.007], use of psychopharmacs [χ^2^(1) = 5.781; p=0.016], and ACEI/ARB medication at discharge [χ^2^(1) = 9.751; p=0.002].

For the three-year mortality outcome, three variables were associated with mortality at three years post-discharge using the independence Pearson Chi-square was applied: previous functional status: [χ^2^(2) = 13.431; p=0.001], use of psychopharmacs [χ^2^(1) = 10.209; p=0.001], and ACEI/ARB medication at discharge [χ^2^(1) = 8.270; p=0.004].

**Table 4:** Continuous variables compared for mortality at one-year and three-year follow-up (Mann-Whitney-U test). BMI: body mass index; SBP: systolic blood pressure.

	One-year mortality		Three-year mortality	
	Mann-Whitney U test	p-value	Mann-Whitney U test	p-value
Age	856	0.625	1409	0.589
Height	390.5	0.418	634	0.237
Weight	432	0.773	736.5	0.85
BMI	852	0.604	1428	0.667
SBP	812	0.414	1464.5	0.829
Creatinine level	592	0.014	1243.5	0.132
Ejection fraction	836.5	0.519	1377	0.459

**Table 5:** Categorical variables compared for mortality at one-year and three-year follow-up (Chi-squared test). NYHA: New York Heart Association; COPD: chronic obstructive pulmonary disease; ACEI/ARB: angiotensin-converting enzyme inhibitors/angiotensin receptors blockers.

	One-year mortality		Three-year mortality	
	Independence Chi-square test (X^2^)	P-value	Independence Chi-square test (X^2^)	P-value
Gender	0.345	0.557	0.000	0.997
Previous functional status	9.978	0.007	13.431	0.001
NYHA class at discharge	12.143	0.007	6.185	0.103
Current smoker	0.729	0.393	0.011	0.918
Diabetes mellitus	0.005	0.943	0.026	0.872
COPD	0.158	0.691	0.744	0.389
Dementia	0.200	0.655	1.482	0.223
Beta-blockers at discharge	0.010	0.921	1.536	0.215
ACEI/ARB medication at discharge	9.751	0.002	8.270	0.004
Use of psychopharmacs	5.781	0.016	10.209	0.001

**Figure 1:**
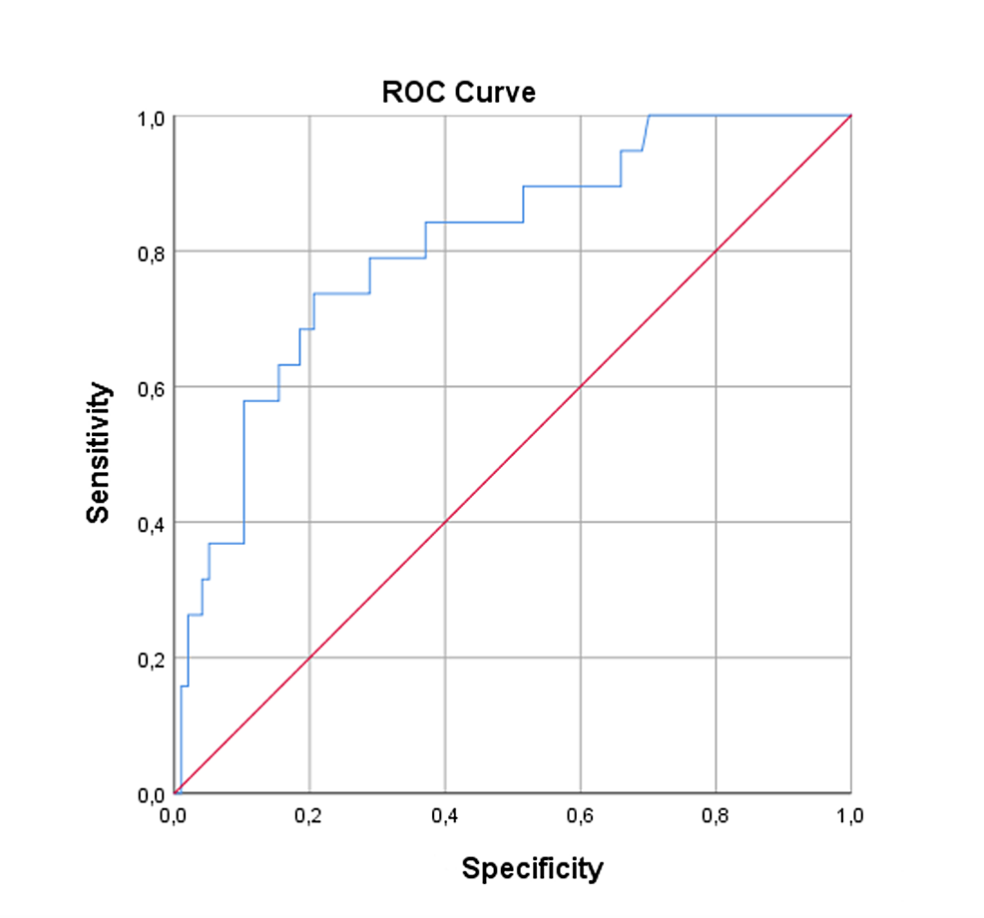
Receiver operating characteristic curve for the one-year mortality model.

Regarding the one-year mortality logistic regression model: 115 patients were included in the analysis; 16.5% of patients (n=19) died during the one-year follow-up. Three variables were retained in the multivariate model and were considered to be important predictor variables: use of psychopharmacs (Wald=6.030, df=1, p=0.014), medication with ACEI/ARB at discharge (Wald=4.524, df=1, p=0.033), and creatininemia at admission (Wald=4.853, df=1, p=0.028). The use of ACEI/ARB at hospital discharge was associated with a lower probability of dying in the first year of follow-up [OR=0.305; 95% CI (0.104-0.895); p=0.031]. The use of psychopharmacs was a strong independent predictor of one-year mortality. Patients medicated with baseline psychopharmacs had a 4.33 times higher probability of dying [OR=4.326; 95% CI (1.449-12.912); p=0.009]. The creatininemia at admission [OR=2.473; 95% CI (1.105-5.536); p=0.028] was also positively associated with mortality in the first year post-discharge, and for each 1 mg/dL increase in creatininemia at hospital admission, the risk of death increased by approximately 2.5 times. To assess the model performance a ROC curve was drawn based on the predictive scoring equation. The area under the curve (AUC) was 0.808 with 95% CI (0.702, 0.913), corresponding to a good discrimination power (Figure 1).

**Figure 2:**
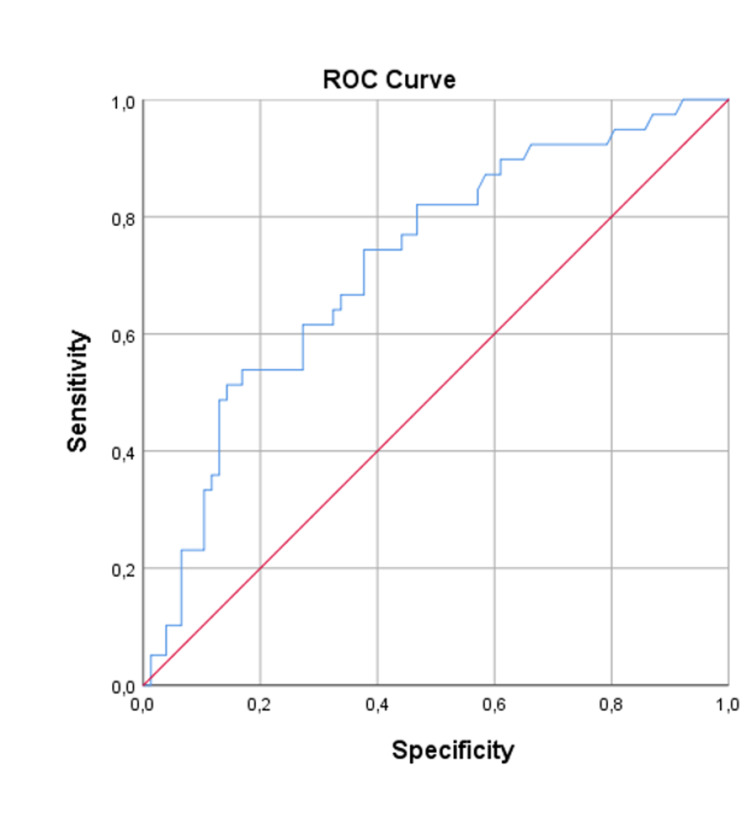
Receiver operating characteristic curve for the three-year mortality model.

In the three-year mortality logistic regression model, 82 patients were included in the analysis and 34 patients are missing data on some of the variables included in the analysis; 49% of patients (n=27) died during the three-year follow-up. Two variables were retained in the multivariate model and were considered to be important predictor variables: use of psychopharmacs (Wald=5.270, df=1, p=0.022) and medication with ACEI/ARB at discharge (Wald=5.587, df=1, p=0.018). The use of ACEI/ARB at hospital discharge was independently associated with a lower probability of dying in the first three years of follow-up [OR=0.285; 95% CI (0.101-0.807); p=0.018]. Comparing with the one-year mortality regression model, the OR is even lower which suggests that the ACEI/ARA benefit increases with time. The use of psychopharmacs was also a strong independent predictor of 3-years mortality. The use of psychopharmacs was positively associated with the probability of dying [OR=3.330; 95% CI (1.192-9.301); p=0.022]. To assess the model performance a ROC curve was drawn. The AUC was 0.720 with 95% CI (0.623, 0.818), corresponding to a moderate discrimination power (Figure 2).

Multivariate logistic regression for one-year and three-year mortality are presented in Table 6.

**Table 6:** Multivariate logistic regression for one-year and three-year mortality. ACEI/ARB: angiotensin-converting enzyme inhibitors/angiotensin receptors blockers.

	One-year mortality Logistic regression model				
	Wald	Df	p-value	OR	95% CI
ACEI/ARB medication at discharge	4.524	1	0.033	0.297	0.097–0.909
Use of psychopharmacs	6.030	1	0.014	4.110	1.330–12.699
Creatinine level at admission	4.853	1	0.028	2.473	1.105–5.536
	Three-year mortality logistic regression model				
	Wald	Df	p-value	OR	95% CI
ACEI/ARB medication at discharge	5.587	1	0.018	0.285	0.101–0.807
Use of psychopharmacs	5.270	1	0.022	3.330	1.192-9.301

## Discussion

This retrospective study represents real-world data that support epidemiologic data, with high mortality after hospitalisation for decompensated HF, observed both at one-year follow-up (16%) and three-year follow-up (34%). Hospital admission for acute decompensated HF is a harbinger of progressive disease, carrying one-year mortality of approximately 30% to 40% and substantial symptom burden [16]. Although advances in management and treatment of HF led to significant improvements in survival, HF prevalence is increasing, and death rates remain high [21]. The population studied was hospitalised in an HF specialised unit, integrated into a structured multidisciplinary HF program, dedicated to a generally younger population, with better functional status, when compared to those normally hospitalised in Internal Medicine wards, justifying a lower mortality.

Variable disease trajectories in HF make prognosis in individual patients very challenging and highly variable [22]. Identifying patient's survival prospects based on risk factors can help define goals of care and target appropriate populations who would benefit from specialised palliative care. The MAGGIC score was useful for predicting mortality in our population, as there was no statistically significant median difference between real mortality compared to the expected three-year mortality calculated by the MAGGIC score.

From the variables studied in this population, previous functional status, NYHA class at discharge, use of psychopharmacs, and creatinine level at admission were statistically significant predictors of the worst prognosis. ACEI/ARB medication at discharge was found to be a protective factor for mortality, with benefits increasing with time.

Heart and brain disorders frequently co-exist, due to having common risk factors and a degree of interaction. In the setting of HF in the elderly, brain disorders, as dementia and depression are very common comorbidities and its management could be challenging. Loss of ability to self-care can lead to very poor QoL [23]. In this study, we verified that the use of psychopharmacs was found to be a strong independent predictor of both one and three years mortality, which can reflect that patients with mental comorbidities had poorer outcomes. Dementia diagnosis was associated with high mortality and worst outcome at three years follow-up though not statistically significant (p=0.223), however, the number of patients with dementia in this population was little, due to the context of a specialised HF unit, dedicated to patients with potential for benefiting a rehabilitation program.

We could verify that previous functional status had a strong impact on the outcome, with poorer functional status associated with higher mortality both at one and three years follow-up (p=0.007 and p=0.001, respectively). This supports previous studies that report that poor functional status is associated with poorer HF outcomes [24].

Congestive signs at discharge are a known predictor of rehospitalizations and mortality [25]. In this study, we proved that higher NYHA at discharge was also associated with mortality at one-year follow-up (p=0.007). In our opinion, patients in NYHA III at discharge are a group who highly benefits from referral for palliative care not only for the high mortality associated but also for the high symptom burden associated with poor QoL.

Diagnosis and management of patients with both kidney disease and HF are complex as their interaction and pathophysiology are bidirectional [26]. The association of HF and chronic kidney disease is well-known and related to increased morbidity and mortality, plus some kidney-specific risk factors (malnutrition, anaemia, myocardial stunning) can lead to decompensated HF and hospitalisation [27,28]. In this study, we were able to demonstrate that higher levels of creatinine at admission were predictive of mortality, namely at one year post-discharge. Furthermore, an increase of 1 mg/dL in creatinine level at admission, was attributed to a 2.5-fold increase in the risk of death. Besides mortality, the interlinked cycle of heart and kidney failure and all the cardio-renal syndrome spectrum is associated with dyspnoea, fatigue, pain, bone and mineral disorders, and depression. All these symptoms should be addressed by a multidisciplinary team in order to promote Qol, reduce emergency visits and hospital admissions [29].

QoL of patients with HF has improved throughout times, with reduced symptoms and increased exercise capacity, as well as lower mortality rates, with the introduction of disease-modifying drugs for reduced ejection fraction and the cumulative benefit of combining treatments [30]. In our study, we verified that patients with ACEI/ARB treatment at discharge were associated with an important independent protector factor, associated with a lower probability of dying in the first three years of follow-up, and its benefit increases with time.

Limitations of this study include the limited size of the population and its characteristics as it was performed at a specialised HF unit with patients with better functional status and better prognosis in general, which might limit the generalizability of the study. Also, it is a retrospective design with all related limitations. Conducting a prospective study including a wider range of variables, like implanted cardiac devices and differentiating between psychopharmacs, could be of great value.

## Conclusions

As the health system evolves, palliative care is becoming essential in the care of patients with HF. It is applicable early in the course of illness, in conjunction with other therapies that are intended to prolong life, and encompasses both general and specialist care. Despite consensus about the need for integration of palliative care in the management of HF, there are still no clear indications on how to implement the guidelines and early select patients who benefit from a specialised approach. During hospitalisations, palliative care needs should be recognised and patients in end of life should be identified. Prognosis in HF is hard to predict, particularly in patients hospitalised with acutely decompensated HF, but risk factors related to poorer outcomes, like decreased functional status and comorbidities such as cognitive impairment and kidney dysfunction, should be considered when setting goals of care and considering specialised palliative care. Patients in NYHA III at discharge are a group that could highly benefit from a palliative care approach, for the higher mortality risk and also for the high symptom burden. The MAGGIC risk score can be a useful tool to predict prognosis and help identify patients who benefit specialised palliative care, however, further studies with bigger populations are needed. Hopefully, this study has achieved its aim to highlight the lack of clear criteria for referral of HF patients and promote research about prognostication based on real-world data.

An individualised patient-centred approach and advance care planning are imperative, based on patient goals and needs, including symptoms and functionality rather than prognosis alone. Hospitalisation should be seen as an opportunity to optimize care for HF patients and integrate palliative care, especially in advanced stages where goals of care change and management of physical, emotional, and spiritual symptoms should become the focus of care, promoting better QoL.
